# Retraction: The impact of different weed management strategies on weed flora of wheat-based cropping systems

**DOI:** 10.1371/journal.pone.0277594

**Published:** 2022-11-16

**Authors:** The PLOS ONE Editors

The *PLOS ONE* Editors retract this article [1] because it was identified as one of a series of submissions for which we have concerns about authorship, competing interests, and peer review. We regret that the issues were not addressed prior to the article’s publication.

MH did not agree with the retraction. MS, KJ, MASR, LW, MAES, and MNA either did not respond directly or could not be reached.
